# Gaining Greater Insight into HCV Emergence in HIV-Infected Men Who Have Sex with Men: The HEPAIG Study

**DOI:** 10.1371/journal.pone.0029322

**Published:** 2011-12-22

**Authors:** Christine Larsen, Marie-Laure Chaix, Yann Le Strat, Annie Velter, Anne Gervais, Isabelle Aupérin, Laurent Alric, Xavier Duval, Patrick Miailhes, Corinne Pioche, Stanislas Pol, Lionel Piroth, Elisabeth Delarocque-Astagneau, for the steering committee of the HEPAIG study

**Affiliations:** 1 National Institute for Public Health Surveillance (InVS), Saint-Maurice, France; 2 University Paris Descartes, EA 3620, Virology Department, APHP, Paris, France; 3 Hepatology Department, Bichat University Hospital, Paris, France; 4 Internal Medicine Department, Community Hospital, Montreuil, France; 5 Digestive Department, Toulouse III University, Toulouse, France; 6 Infectious Diseases Department, Bichat University Hospital, Paris, France; 7 Infectious Diseases Department, Hospices Civiles de Lyon, Croix-Rousse Hospital, Lyon, France; 8 University Paris Descartes, Inserm U1016, Liver Unit, Assistance Publique-Hôpitaux de Paris, Cochin Hospital, Paris, France; 9 Infectious Diseases Department, University Hospital, Dijon, France; 10 Unité d'Épidémiologie des Maladies Émergentes, Institut Pasteur, Paris, France; UCL Institute of Child Health-, University College London, United Kingdom

## Abstract

**Objectives:**

The HEPAIG study was conducted to better understand Hepatitis C virus (HCV) transmission among human immuno-deficiency (HIV)-infected men who have sex with men (MSM) and assess incidence of HCV infection among this population in France.

**Methods and Results:**

Acute HCV infection defined by anti-HCV or HCV ribonucleic acid (RNA) positivity within one year of documented anti-HCV negativity was notified among HIV-infected MSM followed up in HIV/AIDS clinics from a nationwide sampling frame. HIV and HCV infection characteristics, HCV potential exposures and sexual behaviour were collected by the physicians and via self-administered questionnaires. Phylogenetic analysis of the HCV-NS5B region was conducted.

HCV incidence was 48/10 000 [95% Confidence Interval (CI):43–54] and 36/10 000 [95% CI: 30–42] in 2006 and 2007, respectively. Among the 80 men enrolled (median age: 40 years), 55% were HIV-diagnosed before 2000, 56% had at least one sexually transmitted infection in the year before HCV diagnosis; 55% were HCV-infected with genotype 4 (15 men in one 4d-cluster), 32.5% with genotype 1 (three 1a-clusters); five men were HCV re-infected; in the six-month preceding HCV diagnosis, 92% reported having casual sexual partners sought online (75.5%) and at sex venues (79%), unprotected anal sex (90%) and fisting (65%); using recreational drugs (62%) and bleeding during sex (55%).

**Conclusions:**

This study emphasizes the role of multiple unprotected sexual practices and recreational drugs use during sex in the HCV emergence in HIV-infected MSM. It becomes essential to adapt prevention strategies and inform HIV-infected MSM with recent acute HCV infection on risk of re-infection and on risk-reduction strategies.

## Introduction

The overall prevalence of hepatitis C virus (HCV) infection in HIV-infected individuals in France is estimated to be 24% ranging from 93% of those who acquired HIV through injecting drug use to 3% of men who acquired HIV through sexual contact with men [1]. If sexual transmission of HCV is rarely reported among monogamous heterosexual couples [2], outbreaks of acute hepatitis C have been reported since 2000, in HIV-infected men who have sex with men (MSM) who deny injecting drug use [3]–[5], suggesting growing evidence of sexually transmitted HCV in this population. Furthermore, several MSM cohort studies evaluating risk factors associated with HIV transmission have observed an increase in HCV incidence since 2000 among HIV-infected MSM who were not injecting drug users [6]–[9]. Following the French case reports [10], [11], we conducted the prospective HEPAIG study to better understand the transmission dynamics of HCV emergence in HIV-infected MSM and to assess the incidence of HCV infection in this population.

## Methods

### Ethics statement

The study conforms to the ethical guidelines of the 1975 Helsinki Declaration. The study protocol was approved by the French data protection authority. The study's protocol was explained to all patients meeting the case definition who all provided written consent when enrolled.

### Study population

A sampling frame of 290 HIV/AIDS clinics was constructed using the number of HIV/AIDS cases notified in MSM to the national HIV/AIDS case reporting between 2003 and 2005. These clinics distributed throughout France were asked and reminded to report : i) prospectively, each case of acute HCV infection diagnosed in 2006 and 2007 in MSM followed up for HIV infection; ii) at the end of each year during the study period, the total number of cases even in the absence of case.

Acute HCV infection was defined as: 1) a confirmed case when anti-HCV were detected within one year after a documented anti-HCV negativity or when HCV Ribonucleic Acid (RNA) was detected within one year after documented anti-HCV and HCV RNA negativity; 2) a possible case when HCV RNA was detected either: i) within one year after two consecutive documented HCV RNA negatives by PCR; or ii) following clinical signs of hepatitis and alanine aminotransferase (ALT) level over 10 times the upper limit of normal (ULN) within one year after regular controls of normal ALT levels (other causes of acute hepatitis being excluded). All cases were confirmed anti-HCV positive.

Acute HCV re-infection was defined as having detectable HCV RNA following at least two documented undetectable HCV RNA within one year after spontaneous viral clearance (in the absence of anti-HCV treatment) [12] or following at least 24 weeks after the end of anti-HCV treatment [13]. HCV re-infection was confirmed when change in HCV genotype compared with primary infection was observed, otherwise, re-infection was probable.

### Data collection

Two paper-based forms were completed after consent: i) the medical questionnaire completed by the physicians according to the patient responses and from the medical record, collected reasons for HCV screening and data on HIV and HCV infection characteristics, sexually transmitted infections (STI) and potential at-risk exposures (drug use, tattoo/piercing, medical invasive procedures, unprotected sex) within the twelve months before acute HCV infection diagnosis; ii) the self-administered questionnaire, completed secondarily (e.g. at home) by the patient and returned by post to the main investigator. With 68 wide-ranging questions, this questionnaire collected data on socio-demographic characteristics, social and sexual life. Specifically, questions were focused on the six-month period before HCV diagnosis and assessed medication consumptions, snorting or injecting drug use, history of sexually transmitted infection (STI) and sexual behaviour with the regular and casual partners (if any) including number of male sexual partners, type of sexual practices (e.g. unprotected sex, fisting, bondage, discipline, sadism, masochism (BDSM), drug use during sex including type of drug and mode of administration) and sex venues attendance.

HCV RNA detection and HCV genotyping were carried out in routine by the clinics' laboratories during the study period. These laboratories used commercially available HCV RNA assays (based on real-time Polymerase Chain Reaction (PCR) technology) and for genotyping the same standardized assays based on reverse hybridisation technology (Inno-LiPA) or direct sequencing (based on real-time PCR technology). Of note, these laboratories were involved in the French panel quality control study [14].

For men who declined to participate, dates of HIV and HCV diagnosis, age at HCV diagnosis, and region of care were collected.

### Phylogenetic analysis

After patient consent, the first-RNA-positive plasma sample available after the diagnosis of acute HCV infection C was centralized in the HEPAIG reference laboratory and stored at −80°C. HCV RNA was extracted using the Qiamp viral RNA method (Qiagen, Courtaboeuf, France). The primers used for the HCV-NS5B amplification were: outer primers (S0 755: 5′-TATGAYACCCGCTGYTTTGACTC-3′; ASO 1121: 5′-GCNGARTAYCTVGTCATAGCCTC-3′) and inner primers (S0 755: 5′-TATGAYACCCGCTGYTTTGACTC-3′; ENO2: 5′-GCTAGTCATAGCCTCCGT-3′) for the hemi-nested PCR. Primers were designed to amplify a 382 bp c-DNA fragment of NS5b. After purification of the PCR products, direct sequencing was performed as previously described [11]. All sequences were aligned with Clustal X 2.0.11 ® software. Pairwise evolutionary distances were estimated with DNADist, using Kimura's two-parameter method, then the phylogenetic trees were constructed by a neighbour joining method (neighbour program implemented in the Phylip package) [15]. The reliability of each tree topology was estimated from 1000 bootstrap replicates [15].

Reference sequences used for comparison were retrieved from the Los Alamos HCV Database (6bD84262, 7bD84263, 6aY12083, 11aD63822, 8bD84264, 9aD84265, 3aD17763, 3aD28917, 10aD63821, 3bD49374, 4aY11604, 1aM62321, 1aM67463, 1g_Egypt, 1bM58335, 1bU45476, 1bD13558, 1cD14853, 5aAF064490, 5aY13184, 2bD10988, 2cD50409, 2aD00944, 1a004102, 3a.AF046866 , 3a.D28917, 3a.009824) (http://hcv.lanl.gov). Eleven local NS5B genotype 4d sequences from French patients were added: FrSSD41, FrSSD85, FrSSD58, FrSSD37, FrSSD65, FrSSD50, FrSSD171, FrSSD163, FrSSD164, MRS95, P9_RCP, 7 of them were identified in IVDU (FrSSD41, FrSSD171, FrSSD085, FrSSD092, FrSSD163, FrSSD164, FrDSS050). Additional sequences of genotypes 1a and 3a were also included in the analyses (four genotype 1a sequences from French patients (AJ231494, AJ231491, FrSSD54, FrSSD27), 1.AJ851228, 1a.EU155345 to 1a.EU155349, 1a.EU482853, 3a.GQ332555 to 3a.GQ332557, 3a.X76918).

Sequences reported in this study (n = 32) have been submitted to GenBank and can be retrieved under accession numbers JF964963 to JF964994.

The HCV genotyping results taken into account for the purpose of the analysis were all of those from the HEPAIG reference laboratory and, if not available, those from the clinic' laboratories.

### Statistical analysis

Individuals included in the sample were considered to have been randomly sampled by a two-stage cluster design. An inclusion probability was calculated for each individual, equals to the inclusion probability of the participating clinic. Then sampling weights were calculated equal to the inverse of the inclusion probabilities. HCV incidences were estimated using the Horvitz-Thompson estimator including sampling weights and widely used in the classical sampling literature. Estimates were improved by a post-stratification technique using census data (number of HIV-infected MSM followed up annually in each clinic, by year). Estimated variances associated to HCV incidence estimates were also calculated. Assuming a normal distribution for incidence estimates, 95% confidence intervals (CI) were then computed. To estimate HCV incidence, the estimated number of acute HCV infection cases was divided by the total number of HIV-infected MSM followed up annually in the participating clinics. Data were analyzed using STATA® 11.0 (StataCorp, College Station, TX). Fisher's exact test, χ^2^ test, Student's *t* test and Wilcoxon's test were used. Values of *P* value<0.05 were considered statistically significant.

## Results

### Incidence of HCV infection in HIV-infected MSM

In 2006 and 2007, respectively, 99 and 96 clinics that notified 80% of the new AIDS diagnosis in MSM in France in 2003 reported 56 and 46 cases of acute hepatitis C in HIV-infected MSM (64% in the Parisian region). This corresponds to an estimated number of 110 and 87 cases, and to an estimated HCV incidence of 48/10 000 [95% CI: 43–54] in 2006 and, 36/10 000 [95% CI: 30–42] in 2007.

### Patient clinical characteristics

Of the 102 MSM with acute HCV infection, 80 agreed to participate. Compared with the participants, the nonparticipants were older at HCV diagnosis (mean age: 44.0 years versus 40.8 years; *P* = 0.03), but did not differ according to region of care, year of HCV diagnosis, and time between HIV and HCV diagnosis.

Of the 80 men who agreed to participate, 48 (60.0%) sought care in the Parisian region (Table 1). Median age at HCV diagnosis was 40 years (inter-quartile range (IQR): 36–44). At the time of the acute HCV infection diagnosis, 44 men (55.0%) were HIV diagnosed before 2000, 8 men (10.0%) had a previous diagnosis of AIDS-defining illness, 14 (17.5%) had CD4 cell counts below 350/mm3 and 24 (30.0%) had no antiretroviral treatment. HCV infection was diagnosed because of an elevated ALT level in 62 men (77.5%) with concomitant diagnosis of STI in 29 MSM (syphilis in 20; Chlamydia rectal infection in 8; gonorrhea in one). At the time of diagnosis or in the year before HCV diagnosis (medical questionnaires), overall, 45 men (56.2%) had at least one STI (syphilis for 34 men) and, 22 (27.5%) had not (no information: 13 men) (Table 2). Regarding information on potential exposures to HCV in the year prior HCV diagnosis collected by the physicians, no exposure was reported for 60% of the MSM and snorting drug use but no injecting for 26% (Table 2),

**Table 1:** Main characteristics of the 80 HIV-infected MSM at acute HCV infection diagnosis reported by physicians, medical questionnaire, HEPAIG study, 2006–2007.

Region of care	Paris area	48 (60.0)
Age, years		40 (36–44)
Year of HCV diagnosis	2006	41 (51)
	2007	39 (49)
Year of HIV diagnosis	<1996	30 (37.5)
	1996–1999	14 (17.5)
	≥2000	36 (45.0)
Time between HIV and HCV diagnosis, years		9 (3–13.5)
HIV clinical stage	primary infection	2 (2.5)
	asymptomatic	58 (72.5)
	symptomatic	12 (15.0)
	AIDS-defining illness	8 (10.0)
HIV viral load	undetectable^a^ under HAART	46 (57.5)
	detectable without HAART	22 (26.2)
	detectable under HAART	10 (12.5)
	missing	2 (2.5)
CD4 cell count/mm^3^	>500	46 (57.5)
	350–500	20 (25.0)
	200–349	11 (13.7)
	<200	3 (3.8)
Circumstances of HCV diagnosis^b^	systematic anti-HCV screening	17 (21.2)
	increased ALT level	62 (77.5)
	at risk behaviour	27 (33.7)
	jaundice	9 (11.2)
Concomitant diagnosis of STI	yes	29 (36.2)
	no	43 (53.8)
	missing	8 (10.0)
Acute HCV infection	confirmed^c^	59 (74.0)
	possible^d^	16 (20.0)
	re-infection^e^	5 (6.0)
ALT level, xULN	≤1	6 (7.5)
	>1–<3	10 (12.5)
	3–5	14 (17.5)
	>5–7	8 (10.0)
	>7–10	4 (5.0)
	>10	35 (43.7)
	not available	3 (3.7)
HCV viral load,log10, IU/mL		5.9(5.1–6.5)
HCV genotype	1	26 (32.5)
	3	8 (10.0)
	4	44 (55.0)
	not available	2 (2.5)

Note. Data are no. (%) of patients or median values (inter-quartile range); MSM, men who have sex with men; HAART, highly active antiretroviral treatment; STI, sexually transmitted infection; ALT, alanine aminotransferase; ULN, upper limit of normal; ^a^<50 copies/mL; ^b^several possible answers; ^c^anti-HCV (+) within one year after anti-HCV (-) or HCV RNA (+) within one year after both documented anti-HCV (-) and HCV RNA (-); ^d^HCV RNA (+) either: i) within one year after two documented HCV RNA (-), or, ii) with symptoms of acute hepatitis and ALT level >10xULN within one year after regular controls of normal ALT (other causes excluded); ^e^possible acute HCV infection following primary infection with confirmed viral clearance.

**Table 2:** Possible HCV exposures and sexually transmitted infections in the year preceding acute HCV infection diagnosis in the 80 HIV-infected MSM, medical questionnaire, HEPAIG study, 2006-2007.

		n (%)
Possible exposures to HCV^*^	Occupational exposure	1 (1.3)
	Tattoo/piercing	3 (3.8)
	Mesotherapy, acupuncture, varicose vein sclerotherapy	3 (3.8)
	Surgery, digestive endoscopy	11 (13.7)
	Snorting drug use	21 (26.3)
	Injecting drug use	0
Sexually transmitted infection*	Syphilis	34 (42.5)
	Chlamydia rectal infection	14 (17.5)
	Genital herpes	5 (6.3)
	Gonorrhoea	4 (5.0)
	Anal HPV infection	2 (2.5)

Note. MSM, men who have sex with men; HPV, Human Papilloma virus; *several possible answers.

Acute HCV infection was confirmed in 59 MSM (74.0%) and possible in 21 (including re-infection in 5). Jaundice was reported for 9 men (11.2%) (Table1). ALT level was normal in six MSM and HCV viral load was below 5.9 log_10_ IU/mL in 39 men (48.8%). HCV genotypes were mainly genotype 4 (55.0%) and 1 (32.5%), but this distribution was different between 2006 and 2007, genotype 4 decreasing from 70.7% [95%CI = 59.6−79.8] to 38.5% [95%CI = 28.2−49.8] (*P* = 0.004), and genotype 1 increasing from 19.5% [95%CI = 12.1−29.9] to 46.1% [95%CI = 35.3−57.4] (*P* = 0.01).

### Potential exposures reported by the participants (self-administered questionnaire)

Fifty three men (66%) returned the confidential self-administered questionnaire. When comparing their medical questionnaires, the 27 who did not complete the self-administered questionnaire presented similar socio-demographics, HIV and HCV infection characteristics, and history of STIs and of potential HCV exposures. Among these 53 MSM, the median number of sexual partners was 20 (IQR: 10–30) in the six months before HCV diagnosis, 40 men (75.5%) sought their partners online, and 42 (79.2%) at sex venues. Among the 27 men (50.9%) who had a regular sexual male partner, 26 reported having sex in group in the six months before acute HCV infection. Overall, 49 men (92.4%) reported having sex with casual sexual partners who were HIV-infected in 81.6%. Sexual practices with casual partners were predominantly unprotected (Table 3) and included fisting in 35 (71.5%), BDSM in 26 (58.1%) and, bleeding during sex was reported by 26 men (58.1%). In the six months before HCV diagnosis, 28 men (52.8%) were on psychiatric medications including antidepressant for 8 (Table 4).

**Table 3:** HIV and HCV serologic status of sexual male partners and sexual practices with regular and casual partners in the six months before acute HCV infection diagnosis in HIV-infected men who have sex with men (self-administered questionnaires, HEPAIG study, 2006–2007).

		Regular sexual partners	Casual sexual partners
		n = 27	n = 49
HIV serologic status	positive	20 (74.0)	40 (81.6)
	negative	7 (26.0)	-
	unknown	0	9 (18.4)
HCV serologic status	positive	9 (33.3)	3 (6.1)
	negative	14 (51.9)	-
	unknown	4 (14.8)	46 (93.9)
Sexual practices	insertive anal sex	19 (70.4)	42 (85.7)
	*unprotected*	*18 (94.7)*	*36 (85.6)*
	receptive anal sex	25 (92.6)	48 (98.0)
	*unprotected*	*24 (96.0)*	*43 (89.6)*
	fisting	15 (55.6)	35 (71.4)
	*unprotected*	*10 (66.7)*	*20 (57.1)*
	BDSM		
	never	12 (44.4)	23 (46.9)
	rarely	8 (29.6)	18 (36.7)
	often	7 (25.9)	8 (16.3)
Bleeding during sexual practices		15 (55.6)	23 (46.9)

Note. Data are no (%) of patients; BDSM, bondage, discipline, sadism, masochism.

**Table 4:** Drugs consumptions in the six months before acute HCV infection diagnosis in the 53 HIV-infected MSM with self-administered questionnaire, HEPAIG study, 2006–2007.

Psychoactive treatment^a^	no	23 (43.4)
	yes	28 (52.8)
	not available	2 (3.8)
Drug use during sex^b^	Amyl nitrite	47 (88.7)
	*regular use* ^c^	*29 (61.7)*
	Marijuana	22 (41.5)
	*regular use*	*11 (50.0)*
	Ecstasy	21 (39.6)
	*regular use*	*4 (19.0)*
	Cocaine	21 (39.6)
	*regular use*	*6 (28.6)*
	*Intra-rectal use*	*5 (23.8)*
	*injecting use*	*1 (4.8)*
	Heroin	0
	Crystal meth/speed/MDA/other methamphetamines	7 (13.2)
	*regular use*	*1 (14.3)*
	*Intra-rectal use*	*2 (28.6)*
	*Injecting use*	*0*
	GHB	25 (47.2)
	*regular use*	*8 (32.0)*
	Ketamine	11 (20.7)
	*regular use*	*1 (9.1)*

Note. Data are no. (%) of patients. MDA, 3–4 methylene dioxy-amphetamine; GHB, gamma hydroxy butyric acid; ^a^including anxiolytic, hypnotic or antidepressant; ^b^several possible answers; ^c^use at least 10 times during the six-month period

Injecting use of cocaine (with sharing syringes) was reported by one patient G1-infected. During sex, poppers (amyl nitrite) were used by 47 men (88.7%) and other recreational drugs (including: marijuana, ecstasy, cocaine, γ-hydroxybutyric acid (GHB), ketamine, and methamphetamine) by 33 (62.3%). At least two different recreational drugs (not including marijuana and poppers) were consumed during sexual practices by 24 men (45.3%). Of the 21 MSM who reported snorting cocaine during sex, 16 reported sharing straws. Intra-rectal use of recreational drugs was reported by nine men, six of whom were G1-infected. Overall, four men (7.5%) reported neither recreational drug use (besides marijuana or amyl nitrite), nor bleeding during sex or fisting or BDSM. For three of these men no STI was diagnosed in the year before HCV diagnosis.

### Phylogenetic analysis

**Figure 1:**
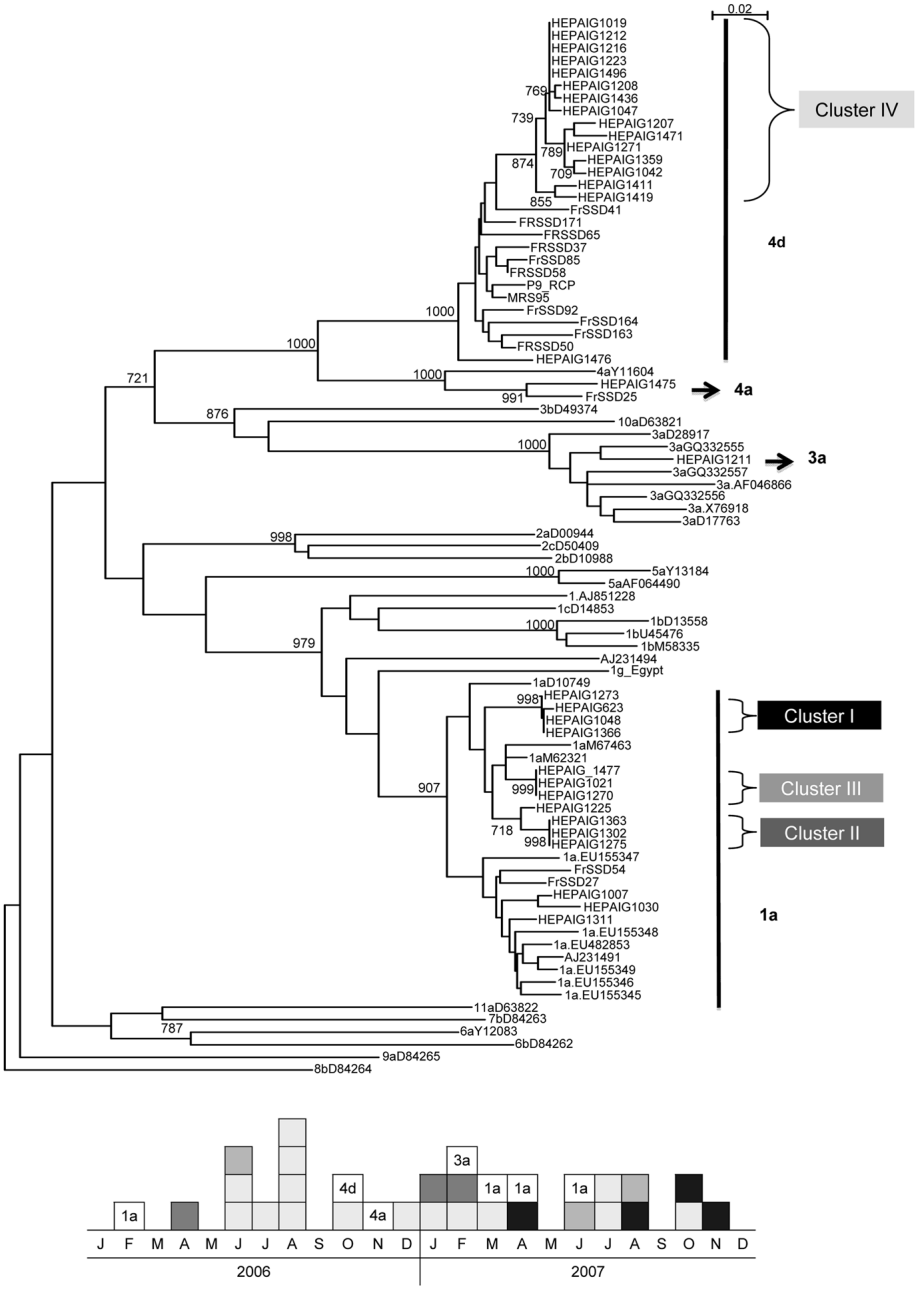
Phylogenetic analysis of the NS5B sequences characterized in 32 HIV-infected MSM with acute HCV infection from 27 reference strains identified with their GenBank reference number (HEPAIG study, 2006–2007, France). Eleven local NS5B genotype 4d sequences from French patients were added: FrSSD41, FrSSD85, FrSSD58, FrSSD37, FrSSD65, FrSSD50, FrSSD171, FrSSD163, FrSSD164, MRS95, P9_RCP including 7 identified in injecting drug users (FrSSD41, FrSSD171, FrSSD085, FrSSD092, FrSSD163, FrSSD164, FrDSS050). Additional sequences of genotypes 1a and 3a were also included in the analyses (four genotype 1a sequences from French patients (AJ231494, AJ231491, FrSSD54, FrSSD27), 1.AJ851228, 1a.EU155345 to 1a.EU155349, 1a.EU482853, 3a.GQ332555 to 3a.GQ332557, 3a.X76918). Numbers at the node indicate bootstrap values.

No difference was found in terms of socio-demographics or characteristics of HIV and HCV infection between the 32 men with available frozen plasma samples included in the phylogenetic analysis and the others. HCV subtypes 1a (14, 43.7%) and 4d (16, 50.0%) were predominant and, HCV subtypes 3a and 4a each infected one MSM. The phylogenic tree of NS5B sequences showed four clusters: three clusters of subtype 1a (cluster I-III) and one cluster of subtype 4d (cluster IV) with monophyletic 4d sequences from MSM clearly distinct from 4d sequences obtained in French injecting drug users. Similarly, the three clusters of subtype 1a (cluster I-III) are monophyletic and no other sequences of genotype 1a (including sequences from French patients) were included in the three clusters (Figure 1).

Several potential HCV exposures were shared between the fifteen MSM in cluster 4d-IV: at least one STI in the year preceding HCV was diagnosed in ten, eight reported at least one recreational drug use during sex (ecstasy, cocaine, ketamine or GHB) but none reported methamphetamine use and six reported fisting. The MSM of clusters 1a shared sex venues attendance and bleeding during sex (cluster 1a-I and 1a-III) or sex venues attendance and fisting (cluster 1a-II).

### HCV re-infections

Acute re-infection was diagnosed and confirmed in five MSM. The first HCV infection was diagnosed after 2000 and after HIV diagnosis in four. All but one were infected secondarily with HCV genotype 4, all being primarily infected with another genotype (unknown for one who cleared HCV spontaneously after cardiac surgery in childhood). No potential HCV exposure was found for one patient, two had at least one STI in the six months preceding re-infection, two reported unprotected fisting, one reported bleeding during sex and one snorted drugs.

## Discussion

### Incidence

The HEPAIG study, based on a nationwide sampling of infectious diseases and internal medicine departments caring for HIV-infected patients, provides estimates of the incidence of acute HCV infection in the population of HIV-positive MSM in France. HCV incidence estimates were below 0.5% and remained stable between 2006 and 2007. Since 2002, the French expert group for medical management of HIV infection has recommended that HCV screening be repeated periodically, particularly for patients reporting injecting drug use. After the first reports of HCV outbreaks in HIV-positive MSM described in 2004 [10], [16], HCV screening at least once a year has been strongly encouraged for HIV-infected MSM. The median level of ALT at diagnosis and proportion of jaundice in our study (11%) were as expected for HIV-positive individuals with recently acquired HCV infection [17]. These estimates are similar when compared to the two European MSM cohort estimates [8], [9] calculated retrospectively for the 2000–2004 period among HIV-infected MSM who denied injecting drug use, but three times lower than the estimate found in 2006 among HIV-infected MSM attending a public sexual health clinic in England [18]. However, our estimates could be underestimated: i) superinfections (i.e. subsequent infection with a heterologous HCV strain in the presence of a previous HCV strain) that were previously described in HIV-positive MSM [19] have not been included in the HEPAIG study case definition (of note also not part of the 2010 European consensus criteria) [20]; ii) although most of the HIV-infected patients sought care at least once a year for follow-up medical care in hospital department, we may have missed cases who did not.

### At risk exposures

Following the first reports suggesting possible sexual transmission of HCV through traumatic sexual practices in HIV-positive MSM without exposure to injecting drug use [5], [10], two case control studies including HIV-positive MSM patients with a diagnosis of acute HCV infection specifically exploring sexual practices have so far been published [3], [21]: Danta *et al.* recruited patients who had been diagnosed between 1999 and 2005 and suggested that traumatic sexual practices were even riskier in the context of group sex participation. As HCV RNA has been detected in semen and more often in the semen of HIV-infected men [22], they suggested that mucosal traumatic sexual practices could favor HCV transmission; Schmidt *et al.* recruited HIV-positive MSM who had been diagnosed with acute HCV since 2000 and identified risk patterns including fisting, rectal trauma with bleeding, group sex and snorting drugs. The HEPAIG MSM had different possible routes of HCV transmission during sex in the six months before HCV diagnosis: more than one third reported sniffing cocaine with possible sharing of contaminated straws [23], [24], and one of them also reported injecting cocaine; a quarter of those who sniffed cocaine also reported intra-rectal use that may contribute to mucosal damage; over half of the MSM had STI including rectal ulcerative infections [5] and reported BDSM that could lead to exposure to bleeding caused by intimate partner traumatic practices [25]; finally, the use of recreational drugs during sex (reported by more than two thirds of the HEPAIG MSM) that has effects such as disinhibition and unawareness could allow traumatic practices as reflected by the high proportion of MSM (nearly 48%) reporting bleeding experiences.

The HEPAIG study design did not include any control group. Indeed, there was a need for greater insights into potential HCV at-risk practices in the presumed contamination period that could be achieved through a very detailed self-administered questionnaire (at least one hour to complete), we were concerned by the likelihood of a high proportion of control patients who would decline their participation, thus introducing bias in our results. It was also essential to explore potential exposures in the presumed period of contamination among the MSM population not recruited through specific sites likely to be related to potential exposures (e.g. STI clinics) and on nationwide basis.

We compared the HEPAIG MSM to the HIV-infected participants to the large “Net Gay Baromètre” survey conducted via French websites for men as the HEPAIG MSM also sought sex partners on line [26]. The HEPAIG MSM had more sexual partners, more often practiced fisting (71.6% versus 46%) and BDSM (53% versus 27%), had more frequent unprotected anal sex (90% versus 73%) and more often took GHB (47% versus 25%) and ketamine (21% versus 8.3%).

### Phylogenetic analysis

In contrast with the recent cases of acute sexually transmitted HCV infection in HIV-infected MSM reported in Australia and the US, where genotype 1a or 3a were predominant [27], [28], European data on HCV sequencing in HIV-positive MSM showed the predominance of genotype 4d and evolutionary analysis suggested the strains introduction from injecting drug users (IDU) [29]. The HEPAIG sequencing analysis confirmed the predominance of genotype 4d (with 50%) over two years and, showed a monophyletic group of 4d sequences clearly distinct from 4d sequences obtained from injecting drug use population, and even from French injecting drug users. Interestingly, the proportion of genotype 1a (43.7%) was also quite high and increased between 2006 and 2007. Genotype 1a commonly identified in IDU [30] may have been introduced into the MSM population through drug use. In the three clusters 1a, we could identify similar potential exposures to HCV shared between the MSM and these exposures were related to sexual activity whereas for cluster 4d in which more patients were included, multiple and different possible exposures at risk were observed and, none of them were shared between all of the MSM. Indeed, one of the limitations of the phylogenetic analysis is that we could not include all of the patients' isolates. However, we showed that no difference in terms of socio-demographics and characteristics of HIV and HCV infection were found between the MSM who were included in the phylogenetic analysis and those who were not.

### Reinfection

Among the HEPAIG MSM, we documented five cases of HCV re-infection, three of them after sustained virologic response to treatment. Few cases of HCV re-infection in HIV-positive MSM have been reported so far [31], [32] until recent data from the Netherlands demonstrating a high incidence rate of HCV re-infection among HIV-infected previously treated for primary HCV infection [33]. However, most of the first report of re-infections were documented in HIV-negative IDU after treatment or spontaneous clearance [34], [35]. Spontaneous HCV clearance is less likely to occur in HIV-infected patients (10 to 15%) [19], [36] than in HIV-negative IDU (25%) [37], as HIV affects the immune responses to HCV [38]. Also, the opportunity for HCV exposure may be low in comparison to IDU: at risk practices involve less frequent exposure to blood than sharing syringes/injection paraphernalia; HCV prevalence is lower in HIV-positive MSM (3–6%) than in IDU (75–90%) [1], [39] although the true figure in the specific core group of HIV-positive MSM engaged in traumatic sexual practices and recreational drugs use during sex may be higher than in the overall population of HIV-positive MSM.

The HEPAIG MSM were possibly unaware of their own risk-taking towards HCV infection, even though they were probably well-informed on risks of STI through unprotected sexual practices and of HCV transmission through injecting drug use.

HEPAIG provided nationwide estimates of incidence of HCV infection among the HIV-infected MSM population, together with phylogenetic analysis of the HCV strains transmitted and comprehensive description of sexual behaviours in the six months preceding acute HCV infection. It emphasizes the role of widespread use of recreational drugs during sex and, engagement in rough sexual practices in the transmission of HCV. Risk of re-infection is also of concern. Thus, in addition to routine and repeated screening for HCV in HIV-infected MSM, there is a need to adapt strategies to prevent HCV transmission and this could be achieved in a joint effort by health care professionals, public health professionals with advocacy groups.
